# Retraction Note: Magnetically retrievable nanocatalyst Fe_3_O_4_@CPTMO@dithizone-Ni for the fabrication of 4*H*-benzo[*h*]chromenes under green medium

**DOI:** 10.1038/s41598-025-02783-5

**Published:** 2025-05-20

**Authors:** Sepideh Bibak, Ahmad Poursattar Marjani

**Affiliations:** Department of Organic Chemistry, Faculty of Chemistry, Urmia University, Urmia, Iran

Retraction of: *Scientific Reports* 10.1038/s41598-023-44881-2, published online 19 October 2023

The Editors have retracted this Article.

After publication, concerns were raised regarding the veracity of the data presented in Figure 13. The Authors provided raw data on request from the Editors. However, the Editors found that the original Raman spectroscopy pattern is different from that presented in the Article. The Editors therefore no longer have confidence in the results and the conclusions of this Article.

Ahmad Poursattar Marjani disagrees with the retraction. Sepideh Bibak did not respond to correspondence from the Editors regarding this retraction.

